# Fibroblasts mediate the angiogenesis of pheochromocytoma by increasing COX4I2 expression

**DOI:** 10.3389/fonc.2022.938123

**Published:** 2022-09-12

**Authors:** Yongxin Mao, Ran Zhuo, Wenming Ma, Jun Dai, Parehe Alimu, Chen Fang, Danfeng Xu, Lei Ye, Weiqing Wang, Fukang Sun

**Affiliations:** ^1^ Department of Urology, Ruijin Hospital, Shanghai Jiao Tong University School of Medicine, Shanghai, China; ^2^ Department of Urology, Huashan Hospital, Fudan University, Shanghai, China; ^3^ Shanghai Institute of Endocrine and Metabolic Diseases, Department of Endocrine and Metabolic Diseases, Ruijin Hospital, Shanghai Jiao Tong University School of Medicine, Shanghai, China

**Keywords:** pheochromocytoma, angiogenesis, COX4I2, fibroblasts, tumor microenvironment

## Abstract

**Objective:**

Our previous work found COX4I2 was associated with angiogenesis in pheochromocytoma. The purpose of this study was to explore the role of COX4I2 in regulating angiogenesis in pheochromocytoma.

**Methods:**

Distribution of COX4I2 was evaluated by scRNA-seq in one case of pheochromocytoma and the findings were verified by immunostaining. COX4I2 was further knocked down in target cells. Changes of angiogenesis-related genes were evaluated by qPCR in target cells.

**Results:**

The scRNA-seq revealed high mRNA expression of COX4I2 in fibroblasts rather than tumor cells. Immunostaining of COX4I2 confirmed its distribution in fibroblasts. Knocking down COX4I2 in NIH3T3 cell line led to significant reduction of angiogenesis-related genes, especially ANG1 and HGF.

**Conclusions:**

Fibroblasts mediate the angiogenesis of pheochromocytoma by increasing COX4I2 expression, possibly by affecting ANG1 and HGF.

## Introduction

Pheochromocytomas are endocrine tumors arising from the adrenal medulla (1, 2). The blood supply of pheochromocytoma is abnormally rich. The surface of tumor is covered with dilated and tortuous vascular network. This pathological change makes pheochromocytoma challenging for surgeons to completely expose the tumor. In our previous study (3), we used high-throughput proteomics to conduct in-depth analysis of pheochromocytoma with or without abundant blood vessels. Mitochondrial cytochrome C oxidase subunit 4 subtype 2 (COX4I2) showed significant up-regulation in the vascular rich group. Furthermore, we found that the protein level of COX4I2 was positively correlated with the microvessel density in pheochromocytoma.

*COX4I2* is the second isoform of subunit IV encoded by cytochrome C oxidase nuclear gene. As a terminal enzyme in the mitochondrial electron transport chain, it is involved in driving oxidative phosphorylation and catalyzing the transfer of electrons from reduced cytochrome C to oxygen (4–7). At present, there are not many studies reporting that *COX4I2* is directly related to angiogenesis. In a study on endometriosis (8), *COX4I2* was found to be targeted by *MLL1* and is essential for the process of endometrial decidualization. Enzymes containing *COX4I2* reduce oxygen affinity, leading to hypoxia in the endometrial microenvironment, which is critical for successful endometrial implantation and angiogenesis. *HIF-2α* expression was increased during decidualization *in vitro*, but knockdown of *COX4I2* blocked *HIF-2α* expression. This study suggests that the expression of *HIF-2α* in the stroma can be regulated by COX4. Furthermore, another study found that increasing the expression of *COX4I2* increased the production of reactive oxygen species associated with angiogenesis (9–11).

Single-cell RNA sequencing (ScRNA-seq) was first reported in 2009, which refers to a new technology for high-throughput sequencing and analysis of RNA at the single-cell level (12). Due to technical limitations, traditional sequencing technology is performed at the multicellular level. The gene expression information obtained is the average signal of the cell population, and the specific gene expression information of individual cell is lost (13–15). ScRNA-seq can independently provide the RNA expression profile of each cell, and study the relationship between gene expression and cell function more precisely and intuitively. It has been widely used in many disciplines. In the tumor microenvironment (TME), in addition to the tumor cells themselves, various types of immune inflammatory cells, fibroblasts, epithelial cells, and various growth factors, enzymes, microvessels and intercellular substances near the tumor area are involved in the occurrence, development, invasion and metastasis of tumors (16–18). The convenience of scRNA-seq is that it can use different marker genes to identify different cell types under TME. In this study, we performed scRNA-seq in one case of pheochromocytoma tissue. By comparing with the expression profile of *COX4I2*, we can better observe its accurate localization in pheochromocytoma. The purpose of this study is to explore the accurate localization and expression of *COX4I2* in pheochromocytoma tissue and to preliminarily verify whether it plays an exact role in angiogenesis in tumor microenvironment.

## Materials and methods

### Tissue preparations

This work was approved by the clinical research ethics committee of Ruijin Hospital. Informed consent was obtained from patients prior to surgery. The pathological diagnosis was confirmed by pathologists.

The fresh tissue specimen was immersed in the preservation solution, and immediately sent for single-cell suspension preparation. The tissue was cut up into small pieces and digested in a shaker with collagenase at 37°C. After digestion, the cell suspension was filtered through a 40-micron cell sieve to filter out the tissues that have not been fully digested. The filtered cell suspension was centrifuged, discarded the supernatant, resuspended with erythrocyte lysate, washed with PBS, and repeated twice. Finally, cell count and viability test were evaluated.

### Single-cell RNA sequencing

The single cell suspension, barcode-containing gel beads and enzyme mixture were combined, and the droplets were wrapped by a microfluid "double cross" cross system to form a water-in-oil microsystem (Gel Bead-In-Emulsions, GEMs). Then, cell lysis was performed in GEMs, the gel beads were automatically dissolved, and a large number of barcode sequences were released at the same time. Then mRNA was reverse transcribed into cDNA, and cDNA was used as a template for PCR amplification. After the cDNA amplification was completed, quality control of the amplified product should be carried out. Finally, after the amplified product passed the inspection, a sequencing library was constructed and sequenced through the Illumina sequencing platform to obtain sequencing data and perform subsequent data analysis. Use t-distributed stochastic neighbor embedding (t-SNE) for dimensionality reduction; use "Seurat" package to identify important clusters.

### Histological evaluation

The pheochromocytoma tissues were fixed with 10% formalin, embedded in paraffin, and sectioned with a thickness of 5 μm. The paraffin embedded samples were dewaxed and rehydrated with dimethylbenzene followed by gradient ethanol. The nuclei were stained with hematoxylin and the cytoplasm was stained with eosin.

Antibodies included COX4I2 antibody (Diluted 1:100, Catalog number: 11463-1-AP, Proteintech, China), Smooth Muscle Actin Polyclonal Antibody (α-SMA) antibody (Diluted 1:100, Catalog number: 14395-1-AP, Proteintech, China) and Anti-Chromogranin A (CgA) antibody (Diluted 1:100, Catalog number: ab220189, Abcam, UK). The sections were incubated with primary antibody overnight in a humidified chamber at 4°C, and then washed with phosphate buffered saline.

Then, immunostaining was performed with UltraSensitive™ SP (Mouse/Rabbit) IHC Kit (Maixin, China) according to its protocol. The sections were incubated with biotin-labeled secondary antibody at room temperature for 10 minutes. Afterwards, diaminobenzidine staining and hematoxylin counterstaining were used. Slices were fixed with neutral gum.

IF was performed with secondary antibody Alexa Fluor 488 nm or 568nm (Thermo Fisher Scientific, USA). The sections were then washed three times in PBS and the nuclei were stained with DAPI (Thermo Fisher Scientific, USA). Finally, the stained sections were observed and photographed with an optical microscope (Olympus, Japan).

### Cell culture

Mouse embryonic fibroblast NIH3T3 cells were cultured in Dulbecco’s modified Minimal Essential Medium (DMEM, Yobibio, China) supplemented with 10% fetal bovine serum (FBS, Gibco, USA) and penicillin/streptomycin (Gibco, USA) in a 37°C, 5% CO_2_ and humid atmosphere.

### RNA interference

The small interfering RNA (siRNA) lentiviral particle packaging used for COX4I2 interference was purchased from Shanghai Genechem Company. Three COX4I2 siRNA and negative control (NC) sequences were designed as followed in **Table 1**. NIH3T3 cells in logarithmic growth phase were digested by trypsin, and a complete medium was used to make a cell suspension of 30000-50000 cells/ml. The cells were inoculated into the culture plate to ensure that the plate amount reached about 15-30% when infected. According to the manufacturer’s instructions, the infected cells should be changed to the conventional medium within 8-16 hours after infection and continued to culture for 48-72 hours.

**Table 1:** Three COX4I2 siRNA and NC (Negative Control) sequences.

Target	Sequences (5ˊ-3ˊ)
KD1	GGCCCTGAAGGAGAAAGAGAA
KD2	CAGCGAGTCTATGTGTTCCCT
KD3	ACGGAAGAACGGAAAGCCCAA
NC	TTCTCCGAACGTGTCACGT

### RNA extraction and qPCR

Total RNA was extracted from NIH3T3 cells by Eastep^®^ Super Total RNA Extraction Kit (Promega, China) according to the manufacturer’s instructions. The concentration and purity of extracted RNA samples were detected by Nanodrop 2000C spectrometer (Thermo Scientific, USA). Then, total RNA was reversely transcribed to cDNA with a reverse transcription Kit (Promega, China). QPCR was then performed on a QuantstudioTM Dx Real‐Time PCR System (Thermo Fisher Scientific, USA) by using SYBR^®^ Green Premix Pro Taq HS qPCR Kit (Agbio, China). The program was run with the following settings: preheated at 95°C for 30 s, denatured at 95°C for 5 s, annealed at 60°C for 30 s. The total of 40 cycles were repeated. The Ct values of the target genes were corrected by using the internal reference Glyceraldehyde-3-phosphate dehydrogenase (GAPDH) of the same sample. The synthesized primers are shown in **Table 2**.

**Table 2:** Primers used for qPCR.

Gene	Primer sequences (5ˊ-3ˊ)	
*GAPDH*	Forward	AGGTCGGTGTGAACGGATTTG
	Reverse	GGGGTCGTTGATGGCAACA
*COX4I2*	Forward	CTGCCCGGAGTCTGGTAATG
	Reverse	CGTAGCAGTCAACGTAGGGG
*VEGFA*	Forward	GCACATAGAGAGAATGAGCTTCC
	Reverse	CTCCGCTCTGAACAAGGCT
*ANG1*	Forward	CACATAGGGTGCAGCAACCA
	Reverse	CGTCGTGTTCTGGAAGAATGA
*ANG2*	Forward	CCTCGACTACGACGACTCAGT
	Reverse	TCTGCACCACATTCTGTTGGA
*HGF*	Forward Reverse	ATGTGGGGGACCAAACTTCTG GGATGGCGACATGAAGCAG

### Statistical analysis

Statistical analysis was conducted using GraphPad Prism software version 8 (GraphPad Software, USA). The relative expression levels of target genes were calculated through the 2^−ΔΔCt^ method. Comparisons were made by two‐tailed Student’s t‐test. P < 0.05 (*), P < 0.01 (**), P < 0.001 (***) and P < 0.0001 (****) were considered statistically significant.

## Results

### ScRNA-Seq found COX4I2 expression mainly in fibroblast

**Figure 1:**
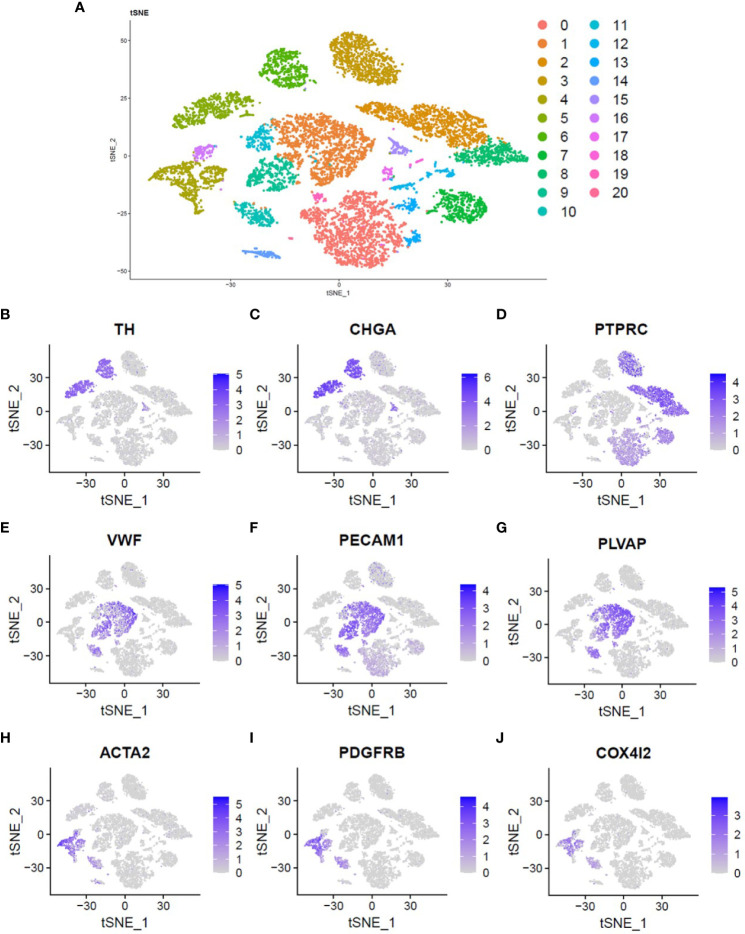
Single cell atlas of pheochromocytoma. **(A)** Two-dimensional t-SNE clustering analysis showed that the cells were divided into 21 clusters and labeled with different colors. **(B, C)** The expression of TH and CHGA genes was used to identify pheochromocytoma tumor cells. **(D)** The expression of PTPRC gene was used to identify immune cells. **(E–G)** The expression of VWF, PECAM1 and PLVAP genes was used to identify endothelial cells. **(H, I)** The expression of ACTA2 and PDGFRB genes was used to identify fibroblasts. **(J)** Location and expression of COX4I2 gene in all cells.

A total of 11,099 cells were obtained. T-SNE found 21 cell clusters (**Figure 1A**). Some cell clusters are described in detail below: Tyrosine hydroxylase (TH) and Chromogranin A (CgA or CHGA) positive pheochromocytoma tumor cells (**Figures 1B, C**), Protein Tyrosine Phosphatase Receptor Type C (PTPRC) positive immune cells (**Figure 1D**), Von Willebrand factor (VWF), Platelet and Endothelial Cell Adhesion Molecule 1 (PECAM1) and Plasmalemma Vesicle Associated Protein (PLVAP) positive endothelium Cells (**Figures 1E–G**), Actin Alpha 2 (ACTA2 or α-SMA) and Platelet derived growth factor receptor B (PDGFRB) positive fibroblasts (**Figures 1H, I**). Interestingly, COX4I2 is highly expressed mainly in fibroblasts (**Figure 1J**).

### COX4I2 staining in pheochromocytoma

**Figure 2:**
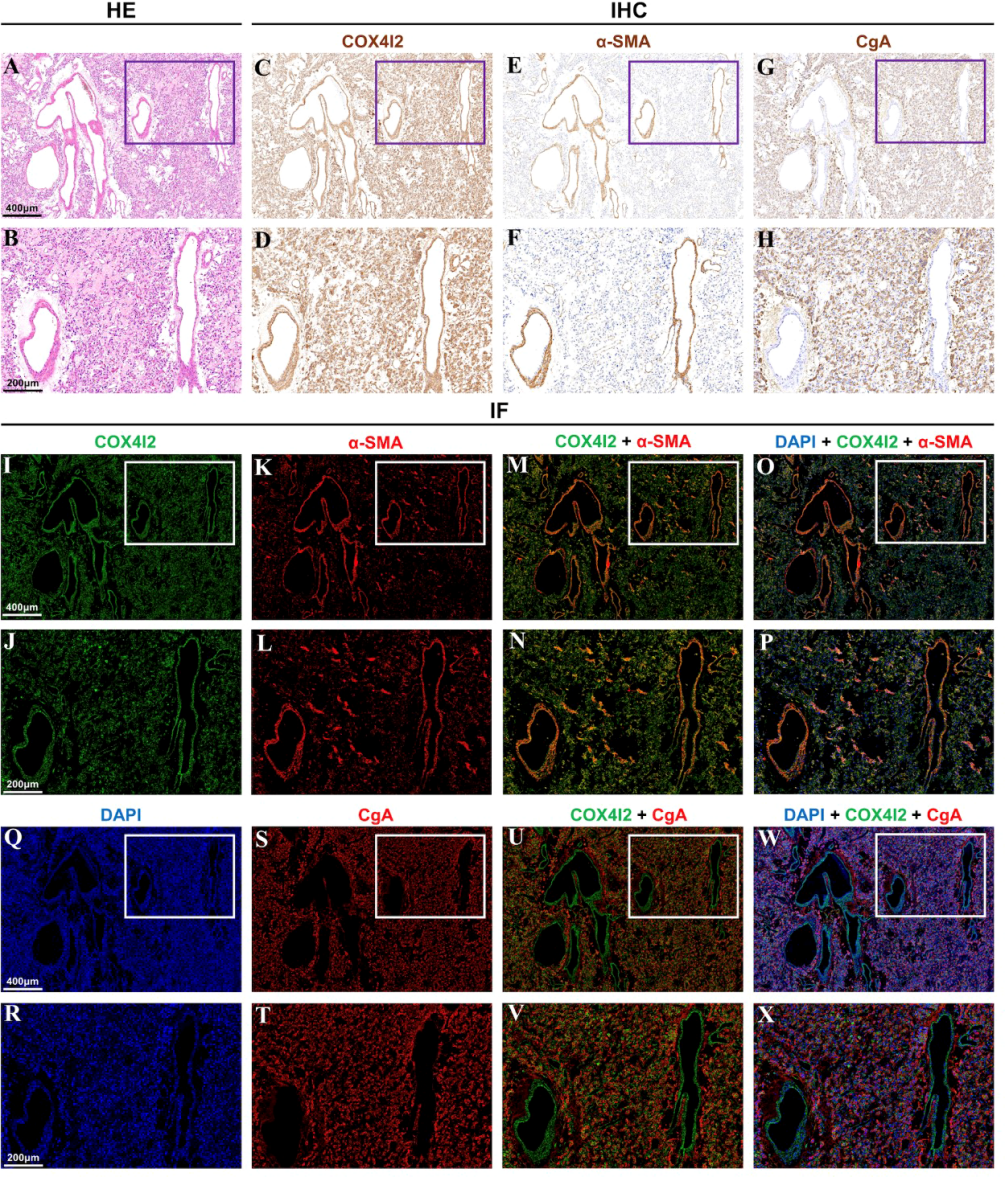
Different staining analysis of the same vascular area in pheochromocytoma tissue. **(A, B)** Hematoxylin and eosin staining showed the vascular lumen area. **(C–H)** Immunostaining of COX4I2, α-SMA and CgA was performed in the same area. **(I–X)**. Immunofluorescence staining of COX4I2, α-SMA, DAPI and CgA was performed in the same area.

Hematoxylin and eosin staining was performed on the pheochromocytoma tissue, as shown in **Figures 2A, B**. This area can clearly see the vascular lumen area of the pheochromocytoma. Immunostaining of COX4I2, CgA and α-SMA were performed in the same area of pheochromocytoma tissue. The results are shown in **Figures 2C–X**. In the vascular lumen area, COX4I2 and α-SMA staining were positive, where CgA staining was negative (**Figures 2C–H, I–L, S–T**). Immunostaining showed that COX4I2 colocalized with α-SMA but not CgA (**Figures 2M–P, U–X**). It can be seen clearly that the co-localized immunofluorescence staining of COX4I2 and CgA in the vascular luminal region of the pheochromocytoma tissue in **Supplementary File Video 1**. This further confirmed the results obtained by the scRNA-Seq analysis.

### Knockdown of COX4I2 led to changes of angiogenesis-related factors in fibroblast cell line NIH3T3

**Figure 3:**
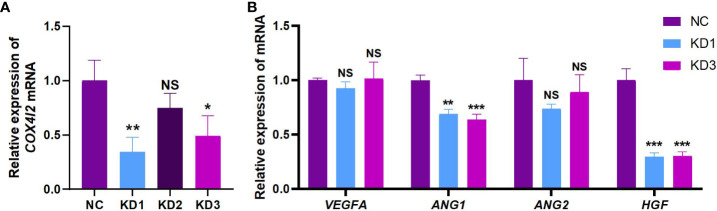
The knockdown of COX4I2 in NIH3T3 cell line resulted in changes of angiogenesis-related factors. **(A)** Changes of COX4I2 mRNA in NIH3T3 cell line after interference of three targets. **(B)** Changes of angiogenesis-related factors VEGFA, ANG1, ANG2 and HGF mRNA in NIH3T3 cell line after COX4I2 knockdown. (NS: No Significance; *: P<0.05; **: P<0.01; ***: P<0.001).

The expression of COX4I2 mRNA in NIH3T3 cell line was interfered by three siRNA target sequences, respectively. **Figure 3A** showed successful knockdown of COX4I2 by KD1 and KD3. With knockdown of COX4I2, the expression of angiogenic related genes in NIH3T3 cell line were reduced. As shown in **Figure 3B**, angiogenic related factors Ang1 and HGF were significantly down-regulated.

## Discussion

Our study firstly showed that COX4I2 is not localized in pheochromocytoma tumor cells, but in fibroblasts in the tumor microenvironment. This important discovery provides us with a clearer direction to study the involvement of COX4I2 in pheochromocytoma angiogenesis, allowing our research team to pay more attention to the tumor microenvironment rather than the tumor cells themselves in subsequent studies. By knocking down the expression of COX4I2 in fibroblasts, the expression of some angiogenic related factors is reduced, indicating that COX4I2 is significantly related to the angiogenesis process.

Angiogenesis is a complex physiological or pathological process involving multiple cells (19, 20). For tumors, the biological behavior of angiogenesis plays an extremely important role in its occurrence and development. Tumor angiogenesis refers to the growth of new capillary blood vessels in the existing capillaries and venules behind the capillaries in the tumor microenvironment (19, 21). It mainly includes the following processes: degradation of vascular endothelial matrix, activation, proliferation, migration of endothelial cells, formation of vascular rings and formation of new basement membrane. On the one hand, more and more studies show that most malignant tumors have dense angiogenesis and rapid growth. The new blood vessel structure of tumor tissue is abnormal and its function is not perfect (22), which provides great convenience for tumor cells to invade the inside of blood vessels and cause distant metastasis with blood flow, leading to a poor prognosis. On the other hand, the new blood vessels of tumors bring a huge challenge to surgery. The new blood vessels increase the possibility of bleeding during the operation, result in excessive blood loss of patients, and at the same time affect the exposure of the surgical field of vision, prolong the operation time and bring inconvenience to the operation.

A previous retrospective study completed by our team showed that in 303 cases of adrenal minimally invasive surgery (23), 62 cases (20%) of adrenal vein anatomical structure with tumor variation were observed, mainly manifested in the formation of new small vein branches or collateral circulation. The venous anatomical variation group had more intraoperative blood loss, longer operation time, longer hospital stays and higher operation cost than the normal anatomy group. Adrenal vein variants are more common in adrenal medullary tumors and larger adrenal masses. Tumor size and the diagnosis of pheochromocytoma are independent risk factors for anatomical variants of adrenal veins. Generally, most pheochromocytomas and paragangliomas were found to be highly vascularized tumors by preoperative imaging and intraoperative observation. In order to conduct a deeper exploration, our team once again implemented high-throughput proteomics research (3). We selected a batch of adrenal pheochromocytoma tissue samples and divided them into a rich blood supply group and a poor blood supply group. Using proteomics detection and analysis, it was found that the differential protein COX4I2 was significantly up-regulated in the pheochromocytoma samples of the rich blood supply group, while the differential protein in the poor blood supply group was PLAT. The results of proteomics were verified in another batch of pheochromocytoma samples by qPCR and immunostaining. In order to further clarify the location of cells and factors in the microenvironment of pheochromocytoma and their roles in tumor angiogenesis, we implemented this study.

Single-cell RNA sequencing technology provides great convenience for understanding the expression and location of COX4I2 in cells of different subtypes in pheochromocytoma tissue. By observing the expression of COX4I2 in different subtypes of cells, we have a clearer grasp of the location of COX4I2 in pheochromocytoma tissue, which suggests that we need to pay more attention to the interaction of various components in pheochromocytoma microenvironment, rather than the tumor cells themselves. Fibroblasts have a great influence on TME through the secretion of cytokines and chemokines, including factors related to angiogenesis (24). Cancer-associated fibroblast (CAF) is an activated form of fibroblast, which is formed by crosstalk between tumor cells and fibroblasts in TME (24). In TME, tumor cells can induce the activation of normal fibroblasts into CAF; on the contrary, CAF secretes related factors to affect the growth of tumor cells. Many studies believe that CAF can promote the formation of tumor blood vessels. First, CAF-derived Platelet-derived growth factor (PDGF) stimulates VEGF production, and the PDGF/PDGFR signaling pathway can regulate tumor angiogenesis; second, CAF can express or recruit angiogenesis-related proteins by itself, directly or indirectly exerting angiogenesis (25, 26).

Both ACTA2 and PDGFR can be used to identify fibroblasts (24). CAF often expresses ACTA2, PDGFR, Fibroblast activation protein (FAP) and vimentin (Vimentin, VIM) and so on. ACTA2, also known as Alpha-smooth muscle actin (α-SMA), is a member of the actin family. It is the most commonly used marker to identify CAF and plays an important role in the maintenance of cell movement, cell structure and integrity. CAF is also called myofibroblasts because of the expression of α-SMA. PDGFR includes two subtypes of α and β, both of which can be used as general markers of fibroblasts. They are mainly distributed on the surface of fibroblasts, astrocytes, neural progenitor cells and pericytes. Overexpression of PDGFR has been observed in multiple tumor types (24). Related studies have pointed out that ACTA2 and PDGFRB have their own advantages and disadvantages as markers for identifying fibroblasts. ACTA2 can better reflect tumor-associated fibroblasts, but it can also be detected in smooth muscle cells and pericytes; PDGFR can be widely expressed in the entire fibroblast population that exists in tumors, and it is not specific for CAF. Therefore, the combined diagnosis of multiple molecular markers is often used in research.

By interfering with COX4I2 mRNA in fibroblasts, we observed a decrease in the expression of ANG1 and HGF, which suggests that COX4I2 may act as an upstream gene of ANG1 and HGF to mediate a pathway of pheochromocytoma angiogenesis. The angiogenin family includes four members, namely Ang1, Ang2, Ang3, and Ang4, which play an important role in tumor angiogenesis independent of the existence of the vascular endothelial growth factor family. Among them, Ang1 and Ang2 are most closely related to angiogenesis. At present, its specific mechanism is not completely clear, but there are increasing researches about members of this family in tumor angiogenesis. Ang1 and Ang2 combine with the ligand Tie2 to activate the Ang/Tie signaling pathway, and then play a role in vascular development, remodeling and stabilization (27). In biological function, Ang1 and Ang2 are slightly different. Ang1 promotes vascular remodeling, maturation and stability by regulating the interaction between endothelial cells and between endothelial cells and surrounding supporting cells. In addition, it can inhibit endothelial cell apoptosis and reduce blood vessel atrophy and degeneration. The main function of Ang2 is to competitively inhibit Ang1 to form unstable blood vessels (28, 29). Hepatocyte growth factor (HGF) was originally named for its ability to stimulate the growth of primary cultured hepatocytes (30, 31). It also plays an important role in the growth and differentiation of cells and some complex biological processes *in vivo*, such as organ formation and angiogenesis. It is a powerful polypeptide growth factor. As one of the cells that can secrete HGF, fibroblasts play an important role in promoting tumor angiogenesis in tumor microenvironment (32). HGF can activate vascular endothelial cells through MAPK and PI3-K pathway, induce the proliferation and migration of vascular endothelial cells, form lumen like structure, and participate in tumor angiogenesis (33, 34).

Both KD1 and KD3 groups successfully knocked down the expression of COX4I2 in NIH3T3 cells, and the KD1 group showed a better knockdown effect. However, compared with the KD3 group, the ANG1 and HGF in the NIH3T3 cells of the KD1 group did not appear more obvious down-regulation, and there was no obvious linear relationship between them. This may be because COX4I2- mediated angiogenesis is complex and diverse, not directly through a single pathway, but under the regulation of multiple factors, resulting in the down-regulation of angiogenesis- related factors. Its deeper mechanism is still worth our follow-up further study.

## Conclusion

In conclusion, fibroblasts mediate the angiogenesis of pheochromocytoma by increasing COX4I2 expression, possibly by affecting ANG1 and HGF.

## Data availability statement

The original contributions presented in the study are publicly available. This data can be found here: https://bigd.big.ac.cn/gsa-human/browse/HRA002817 / HRA002817.

## Ethics statement

The studies involving human participants were reviewed and approved by Clinical research ethics committee of Ruijin Hospital. The patients/participants provided their written informed consent to participate in this study.

## Author contributions

FS, LY and YM: designed the study. FS, WW, JD, CF, DX and LY: treated the patient and collected the data. YM, RZ, WM, and PA: collected and analyzed the data. YM, RZ, and WM: wrote the original draft. LY, WW and FS: reviewed and edited the manuscript. All authors contributed to the article and approved the submitted version.

## Funding

This study was supported by the National Natural Science Foundation of China (81972494) and Scientific Research Project of Shanghai Municipal Health Commission (202040018).

## Acknowledgments

We sincerely thank Shanghai Institute of Endocrine and Metabolic Diseases for the generous gift of mouse embryonic fibroblast NIH3T3 cell line. Thanks to Zhang Jie and Liu Ziyuan for their guidance and help on single-cell sequencing and analysis techniques.

## Conflict of interest

The authors declare that the research was conducted in the absence of any commercial or financial relationships that could be construed as a potential conflict of interest.

## Publisher’s note

All claims expressed in this article are solely those of the authors and do not necessarily represent those of their affiliated organizations, or those of the publisher, the editors and the reviewers. Any product that may be evaluated in this article, or claim that may be made by its manufacturer, is not guaranteed or endorsed by the publisher.

## Supplementary material

Supplementary Video 1 A video that can clearly see the co-localized immunofluorescence staining of COX4I2 and CgA in the vascular luminal region of a pheochromocytoma tissue. COX4I2 antibody was used to reflect the COX4I2-positive region (shown in green), and CgA antibody was used to reflect pheochromocytoma tumor cells (shown in red). The results of immunofluorescence colocalization staining showed that the COX4I2 positive area appeared in the luminal area, but did not overlap with the CgA positive region of the tumor cell region.

## References

[1] Job S Draskovic I Burnichon N Buffet A Cros J Lepine C. Telomerase activation and atrx mutations are independent risk factors for metastatic pheochromocytoma and paraganglioma. Clin Cancer Res (2019) 25(2):760–70. doi: 10.1158/1078-0432.Ccr-18-0139 30301828

[2] Pryma DA Chin BB Noto RB Dillon JS Perkins S Solnes L. Efficacy and safety of high-Specific-Activity I-131-Mibg therapy in patients with advanced pheochromocytoma or paraganglioma. J Nucl Med (2019) 60(5):623–30. doi: 10.2967/jnumed.118.217463 PMC6495236 30291194

[3] Sun F Zhuo R Ma W Yang D Su T Ye L. From clinic to mechanism: Proteomics-based assessment of angiogenesis in adrenal pheochromocytoma. J Cell Physiol (2019) 234(12):22057–70. doi: 10.1002/jcp.28769 31106414

[4] Huttemann M Kadenbach B Grossman LI. Mammalian subunit iv isoforms of cytochrome c oxidase. Gene (2001) 267(1):111–23. doi: 10.1016/S0378-1119(01)00385-7 11311561

[5] Shteyer E Saada A Shaag A Al-Hijawi FA Kidess R Revel-Vilk S. Exocrine pancreatic insufficiency, dyserythropoeitic anemia, and calvarial hyperostosis are caused by a mutation in the Cox4i2 gene. Am J Hum Genet (2009) 84(3):412–7. doi: 10.1016/j.ajhg.2009.02.006 PMC2668012 19268275

[6] Haghjoo N Moeini A Masoudi-Nejad A. Introducing a panel for early detection of lung adenocarcinoma by using data integration of genomics, epigenomics, transcriptomics and proteomics. Exp Mol Pathol (2020) 112 :104360. doi: 10.1016/j.yexmp.2019.104360 31843580

[7] Pajuelo Reguera D Cunatova K Vrbacky M Pecinova A Houstek J Mracek T. Cytochrome c oxidase subunit 4 isoform exchange results in modulation of oxygen affinity. Cells (2020) 9(2):443. doi: 10.3390/cells9020443 PMC7072730 32075102

[8] Wen X Xiong Y Liu H Geng T Jin L Zhang M. Decreased mixed lineage leukemia 1 is involved in endometriosis-related infertility. J Mol Endocrinol (2021) 66(1):45–57. doi: 10.1530/jme-20-0193 33151904

[9] Sommer N Huettemann M Pak O Scheibe S Knoepp F Sinkler C. Mitochondrial complex iv subunit 4 isoform 2 is essential for acute pulmonary oxygen sensing. Circ Res (2017) 121(4):424. doi: 10.1161/CIRCRESAHA.116.310482 28620066 PMC5544581

[10] Jing Y Liu L-Z Jiang Y Zhu Y Guo NL Barnett J. Cadmium increases hif-1 and vegf expression through ros, erk, and akt signaling pathways and induces malignant transformation of human bronchial epithelial cells. Toxicological Sci (2012) 125(1):10–9. doi: 10.1093/toxsci/kfr256 PMC3243743 21984483

[11] Parekh A Das S Parida S Das CK Dutta D Mallick SK. Multi-nucleated cells use ros to induce breast cancer chemoresistance *in vitro* and *in vivo* . Oncogene (2018) 37(33):4546–61. doi: 10.1038/s41388-018-0272-6 29743594

[12] Tang F Barbacioru C Wang Y Nordman E Lee C Xu N. Mrna-seq whole-transcriptome analysis of a single cell. Nat Methods (2009) 6(5):377–U86. doi: 10.1038/nmeth.1315 19349980

[13] Zheng C Zheng L Yoo J-K Guo H Zhang Y Guo X. Landscape of infiltrating T cells in liver cancer revealed by single-cell sequencing. Cell (2017) 169(7):1342. doi: 10.1016/j.cell.2017.05.035 28622514

[14] Carter B Zhao K. The epigenetic basis of cellular heterogeneity. Nat Rev Genet (2020) 22(4):235–50. doi: 10.1038/s41576-020-00300-0 PMC10880028 33244170

[15] Friedman N. Studying gene expression at the level of the single cell. Briefings Funct Genomics (2013) 12(2):73–4. doi: 10.1093/bfgp/elt013 23536214

[16] Anton K Glod J. Tumor-secreted factors that induce mesenchymal stromal cell chemotaxis. Mesenchymal Stromal Cells as Tumor Stromal Modulators (2017):193–214. doi: 10.1016/B978-0-12-803102-5.00008-2

[17] Lorger M. Tumor microenvironment in the brain. Cancers (2012) 4(1):218–43. doi: 10.3390/cancers4010218 PMC3712675 24213237

[18] Shiga K Hara M Nagasaki T Sato T Takahashi H Takeyama H. Cancer-associated fibroblasts: Their characteristics and their roles in tumor growth. Cancers (2015) 7(4):2443–58. doi: 10.3390/cancers7040902 PMC4695902 26690480

[19] Cardenas Delgado VM Nugnes LG Colombo LL Troncoso MF Fernandez MM Malchiodi EL. Modulation of endothelial cell migration and angiogenesis: A novel function for the "Tandem-repeat" lectin galectin-8. FASEB J (2011) 25(1):242–54. doi: 10.1096/fj.09-144907 20876211

[20] Gupta K Zhang J. Angiogenesis: A curse or cure? Postgrad Med J (2005) 81(954):236–42. doi: 10.1136/pgmj.2004.023309 PMC1743249 15811887

[21] Parida S Mandal M. Mechanism of controlling blood vessel growth and development and identification of therapeutics against pathological angiogenesis. In: AttaUrRahman Choudhary MI, editors. Anti-angiogenesis drug discovery and development, vol. 2 . Anti-Angiogenesis Drug Discovery and Development (2014). p. 3–62. doi: 10.1016/B978-0-12-803963-2.50001-6

[22] Ning T Jiang M Peng Q Yan X Lu Z-J Peng Y-L. Low-dose endostatin normalizes the structure and function of tumor vasculature and improves the delivery and anti-tumor efficacy of cytotoxic drugs in a lung cancer xenograft murine model. Thorac Cancer (2012) 3(3):229–38. doi: 10.1111/j.1759-7714.2012.00111.x 28920305

[23] Sun F Zhuo R Ma W He H Ye L Xu D. Retrospective analysis of variant venous anatomy in 303 laparoscopic adrenalectomies and its clinical implications. J Surg Oncol (2019) 119(6):801–6. doi: 10.1002/jso.25364 30697735

[24] Nurmik M Ullmann P Rodriguez F Haan S Letellier E. In search of definitions: Cancer-associated fibroblasts and their markers. Int J Cancer (2020) 146(4):895–905. doi: 10.1002/ijc.32193 30734283 PMC6972582

[25] Gomes FG Nedel F Alves AM Noer JE Chaves Tarquinio SB. Tumor angiogenesis and lymphangiogenesis: Tumor/Endothelial crosstalk and Cellular/Microenvironmental signaling mechanisms. Life Sci (2013) 92(2):101–7. doi: 10.1016/j.lfs.2012.10.008 PMC3740377 23178150

[26] Li M Li M Yin T Shi H Wen Y Zhang B. Targeting of cancer-associated fibroblasts enhances the efficacy of cancer chemotherapy by regulating the tumor microenvironment. Mol Med Rep (2016) 13(3):2476–84. doi: 10.3892/mmr.2016.4868 PMC4768992 26846566

[27] Leligdowicz A Richard-Greenblatt M Wright J Crowley VM Kain KC. Endothelial activation: The Ang/Tie axis in sepsis. Front Immunol (2018) 9:838. doi: 10.3389/fimmu.2018.00838 29740443 PMC5928262

[28] Lemieux C Maliba R Favier J Theoret JF Merhi Y Sirois MG. Angiopoietins can directly activate endothelial cells and neutrophils to promote proinflammatory responses. Blood (2005) 105(4):1523–30. doi: 10.1182/blood-2004-09-3531 15498854

[29] Zheng C Toth J Bigwarfe T MacDougall M Jerath K Bovat K. Non-neutralizing antibodies increase endogenous circulating Ang1 levels. Mabs (2018) 10(8):1260–8. doi: 10.1080/19420862.2018.1521130 PMC6284558 30199300

[30] Nakamura T Nawa K. Ichihara a. Partial purification and characterization of hepatocyte growth factor from serum of hepatectomized rats. Biochem Biophys Res Commun (1984) 122(3):1450–9. doi: 10.1016/0006-291x(84)91253-1 6477569

[31] Yan HL Rivkees SA. Hepatocyte growth factor stimulates the proliferation and migration of oligodendrocyte precursor cells. J Neurosci Res (2002) 69(5):597–606. doi: 10.1002/jnr.10323 12210825

[32] Stoker M Gherardi E Perryman M Gray J. Scatter factor is a fibroblast-derived modulator of epithelial cell mobility. Nature (1987) 327(6119):239–42. doi: 10.1038/327239a0 2952888

[33] Tacchini L Dansi P Matteucci E Desiderio MA. Hepatocyte growth factor signalling stimulates hypoxia inducible factor-1 (Hif-1) activity in Hepg2 hepatoma cells. Carcinogenesis (2001) 22(9):1363–71. doi: 10.1093/carcin/22.9.1363 11532856

[34] Rizwani W Allen AE Trevino JG. Hepatocyte growth factor from a clinical perspective: A pancreatic cancer challenge. Cancers (2015) 7(3):1785–805. doi: 10.3390/cancers7030861 PMC4586794 26404380

